# Biosourced Polymetallic Catalysis: A Surprising and Efficient Means to Promote the Knoevenagel Condensation

**DOI:** 10.3389/fchem.2018.00048

**Published:** 2018-03-27

**Authors:** Pierre-Alexandre Deyris, Valérie Bert, Sébastien Diliberto, Clotilde Boulanger, Eddy Petit, Yves-Marie Legrand, Claude Grison

**Affiliations:** ^1^Laboratoire de Chimie Bio-Inspirée et D'Innovations Ecologiques, UMR 5021 Centre National de la Recherche Scientifique – Université de Montpellier, Grabels, France; ^2^INERIS, Clean and Sustainable Technologies and Processes Unit, DRC/RISK, Parc Technologique Alata, BP2, Verneuil-en-Halatte, France; ^3^Institut Jean Lamour, UMR 7198, Université de Lorraine, Centre National de la Recherche Scientifique, Metz, France; ^4^IEM, Université de Montpellier, Centre National de la Recherche Scientifique, ENSCM, Montpellier, France

**Keywords:** sustainable chemistry, ecocatalysis, bio-sourced catalyst, Knoevenagel condensation, phytoextraction

## Abstract

Zn hyperaccumulator (*Arabidobsis halleri*) and Zn accumulator *Salix* “Tordis” (*Salix schwerinii* × *Salix viminalis*) have shown their interest in the phytoextraction of polluted brownfields. Herein, we explore a novel methodology based on the chemical valorization of Zn-rich biomass produced by these metallophyte plants. The approach is based on the use of polymetallic salts derived from plants as bio-based catalysts in organic chemistry. The formed ecocatalysts were characterized via ICP-MS, X-ray diffraction (XRD), Fourier transform infrared spectroscopy (FT-IR) in order to precise the chemical composition, structure, and behavior of the formed materials. The Doebner-Knoevenagel reaction was chosen as model reaction to study their synthetic potential. Significant differences to usual catalysts such as zinc (II) chloride are observed. They can principally be related to a mixture of unusual mineral species. DFT calculations were carried out on these salts in the context of the Gutmann theory. They allow the rationalization of experimental results. Finally, these new bio-based polymetallic catalysts illustrated the interest of this concept for green and sustainable catalysis.

## Introduction

Over the last decade, there has been an awareness of the elemental sustainability (Hunt et al., 2015). The concerns over the depletion of the traditional sources of elements are actually rising. At the current rate, the known reserves of numerous element such as Zn will be consumed in <50 years (Hunt, 2013). However, elements are not lost but dispersed throughout our environment by human activities. For example, several wastes including municipal solid waste, e-waste, metallurgic, and mining wastes contain elevated levels of metals (Cui and Zhang, 2008; Jung and Osako, 2009). This dispersion is responsible for contamination and therefore degradation of soils. Moreover, this contamination can enter human bodies via inhalation, skin contact, or food chain and may have health impacts. Because of the elemental sustainability and the social issues, the recovery of metals from our waste can be considered as a hazard avoidance and an alternative to primary resources.

In this context, phytoremediation of industrial contaminated soils, a worldwide environmental issue, is a promising method for restoring soils and recovering metal. Certain plants can extract and accumulate metals from the soil into the biomass (van der Ent et al., 2013). This capacity called phytoextraction offers the opportunity to remediate soils (Losfeld et al., 2015). While the metal-enriched biomass might be considered as a hazardous waste, various valorization ways, exploiting recovered metal are currently investigated. Recently, we have studied the development of a series of novel approaches for the recycling and reuse of vital minerals for industrial chemistry, linked with the phytoremediation of contaminated soils on mining sites. Taking advantage of the remarkable ability of certain land plants to hyperaccumulate transition metals into shoots, we have addressed the use of metals derived from contaminated plant waste as Lewis acids, coupling catalysts, oxidizing, and reducing agents in organic synthesis of biomolecules. This was the first chemical recovery of new phytoextraction technologies. Recycling of waste from metal-enriched plants has led to the new concept in chemistry, namely “ecocatalysis” (Grison, 2015). The first results revealed that ecocatalysts (called Eco-M) can be more efficient and selective than traditional catalysts. For example, Eco-Zn were found to be very efficient in the Garcia Gonzalez reaction (Escande et al., 2014c) and showed unusual flexibility in catalyzed preparation of polyhydroxyalkyl furans. The polymetallic systems arising from the biomass produced by phytoextraction are unusual and lead to new associations of chemical species, such as CaMg_2_Cl_6_·12H_2_O, MgP_4_O_11_, or K_2_ZnCl_4_ (Escande et al., 2015a). They exhibit also a better chemo- and regio-selectivity than traditional catalysts in numerous reactions [multicomponent (Grison et al., 2013), domino (Escande et al., 2015d), cycloaddition (Escande et al., 2014b), catalytic reduction (Escande et al., 2015c, 2017b), and oxidation (Escande et al., 2015b, 2017a)]. They can be used as catalysts in synthetic transformations of biomass, leading to molecules of interest to industrial chemicals (perfumes and cosmetics), oligomers of biological interest (Thillier et al., 2013), functionalized aromatic heterocyclic compounds (Clavé et al., 2016, 2017), and building blocks of industrial chemical processes.

In this paper, we wish to carry out a comparative study of new biomass, which derived from two metal-contaminated areas. Both sites are located in the Hauts-de-France region which was heavily impacted by metallurgical activities emissions. As a result, soils and canal sediments were contaminated by metals such as Zn, Cd, Pb, and Cu. For some metal polluted sites, phytomanagement seems to be a relevant option to handle the risks associated to the pollutants and valorize the produced biomass by ecocatalysis (Kidd et al., 2015). Herein, we present two different case studies: a Zn hyperaccumulator *Arabidobsis halleri* (Huguet et al., 2012) and a Zn accumulator *Salix* “Tordis” (*Salix schwerinii* × *Salix viminalis*). *A. halleri* was cultivated on a Zn-contaminated soil for phytoextraction purpose whereas *S. “Tordis”* was planted on a metal contaminated sediment landfill site for bioenergy production. Because both plant species accumulate Zn in a very important amount in their harvestable parts (i.e., leaves), this ability was suitable to ecocatalysis. According to the known optimal zinc accumulation capacity of each plant, the principal objective is to determine if the utilization of these plants is valuable in catalysis. To that end, the catalytic potential of each ecocatalyst was tested by the Knoevenagel condensation as model reaction.

The Knoevenagel condensation is widely used in organic synthesis especially in pharmaceutical industry. For example, it is present in one-step on the synthesis of atorvastatin, pioglitazone, pregabalin, rosuvastatin, or rosiglitazone (Harrington, 2011; Debarge et al., 2014; Vardanyan and Hruby, 2016). The catalysts commonly used are weak organic bases such as pyrrolidine or ammonium salts such as piperidine/acetic acid. Several Lewis acids such as ZnCl_2_, CeCl_3_·7H_2_O-NaI, NbCl_5_, and AlCl_3_ have been reported (Shanthan Rao and Venkataratnam, 1991; Bartoli et al., 2006; Yadav et al., 2009; Li et al., 2012).

## Materials and methods

### Phytomanagement on metal-contaminated brownfield area

#### Biomasses cultivation and collection

The willow cultivar “Tordis” was planted in 2012 in a very short rotation coppice (VSRC) on metal-polluted dredged sediments for the purpose of bioenergy production. The site is located at Fresnes-sur-Escaut (Hauts-de-France, France). The field site was devoted to metal immobilization by using a soil amendment and a soil grass cover and simultaneously to the production of woody biomass by growing willows. The 650 m2 site which contains 350 willows produced ~1.4 tons of dried biomass in optimal conditions. As the willow “Tordis” leaves contained a large amount of Zn, the collection of leaves in November 2015 was undertaken to avoid metal pollution dispersion. At the same time, leaves of the Zn-hyperaccumulator *A. halleri* were collected in a site impacted by a Zn smelter (Auby, Hauts-de-France, France) in December 2015. On this site, numerous *A. halleri* plants are present, as a result of its spread on the entire site from the beginning of the nineteenth century, which allowed the collection of the desired amount of leaves to be processed. *A. halleri* from this site was extensively studied (Gomez-Balderas et al., 2014). Like willows, the management of the Zn-enriched biomass produced on this site was undertaken by exploring the possibility to valorize the plant Zn content.

#### Characterization of biomasses

After homogenization of the collected leaves, a composite sample was formed and split in 10 and five samples for *S. Tordis* and *A. halleri*, respectively. Leaves were oven-dried at 40°C until constant weight. 0.5 g were ground and digested at 180°C for 20 min in 10 mL of nitric acid (67%) and 3 mL of ultra-pure water using a microwave digester (Mars Xpress, CEM). The digested samples were filtered to <0.45 μm (Millipore) and acidified to a pH < 2 for preservation. The analyses of metal concentrations were performed by ICP-AES (Agilent 720ES). Two standard references of plant material (white cabbage “BCR-679,” Community Bureau of Reference and tobacco leaves “CTA-VTL-2”) were included for analytical quality control.

### Catalyst preparation and characterization

#### Preparation of the ecocatalysts

Biomasses (usually 100 g) were first dried into an oven at 80°C and then thermally treated in an oven under air flow. The temperature program was a first gradient from 20 to 350°C in 1 h then 2 h at 350°C and a second one from 350 to 550°C in 1 h then 2 h at 550°C before a slow cooling overnight in the oven to obtain ashes. The resulting powder was subjected to a chemical treatment. In the typical procedure, the ashes (10 g) were added to 100 mL of 6 M HCl solution then heated and stirred at 60°C for 3 h. The reaction mixture was filtered through celite. The resulting yellow solution was refluxed during 5 h and then was evaporated at 80°C. The dry residues were crushed to fine powder in a mortar and placed in an oven at 90°C for 2 days. Purification steps are not mandatory in our process.

#### Preparation of the double salts

The preparation of the double salts was adapted from previously published work (Poddar et al., 2015). KCl and MgCl_2_ or ZnCl_2_ were dissolved separately in analytical grade water in the desired ratio. The resulting solutions were combined and then evaporated at 80°C. The dry residues were crushed to fine powder in a mortar and placed in an oven at 90°C for 2 days.

#### Characterization of the catalysts

##### MP-AES analysis (microwave plasma-atomic emission spectrometer)

The samples were digested in 10 mL of reversed aqua regia [1:2 hydrochloric acid (37%):nitric acid (65%)] under a microwave-assisted digestion (Mileston ETHOS Tauch) with the following program: 20–90°C in 7 min, 90–170°C in 5 min, 170–210°C in 3 min, and then 20 min isothermal at 210°C. Samples were filtered and then diluted to 0.1 mg/L in nitric acid 2.5%.

##### X-ray diffraction analysis

X-ray diffraction (XRD) data measurements on the samples dried at 110°C for 2 h were performed by using a BRUKER diffractometer (D8 advance, with a Cu Kα radiation λ = 1.54086 Å) equipped with a Lynxeyes detector.

##### Pyridine-FT-IR (fourier transform infrared spectroscopy)

FT-IR measurements were carried out with pyridine probe. A PerkinElmer Spectrum 100 FT-IR spectrometer was used for recording the spectra. 10 ± 1 mg of catalyst were introduced in a 10 mL vial under argon. Dichloromethane (2 mL) was added followed by pyridine (100 μL) and the resulting mixture was stirred by a vortex during 1 min. Surplus pyridine was adsorbed, and the samples were degassed for 15 min at 25°C under an argon flux. A sample of the resulting powder (5 ± 1 mg) was picked up in order to record a first spectrum. The samples were then degassed for 25 min at 150°C (10^−3^ Pa) to eliminate the physisorbed pyridine. A second spectrum was then recorded with 5 ± 1 mg of the resulting powder.

#### General procedure for knoevenagel condensations

##### Representative procedure

The catalyst was dried prior to use and was added to an argon flushed 5 mL round bottom flask followed by biphenyl, aromatic aldehyde (5 mmol), and methylene nucleophile (5 mmol). For control experiments, no catalyst was added. After stirring 1 h at 100°C under argon atmosphere, the reaction was cooled under running water and then diluted with ethyl acetate. The conversion of benzaldehyde was estimated by GC-MS analysis using biphenyl as an internal standard. To check the GC-MS calibration, some products were purified by flash-chromatography using ethyl acetate/cyclohexane as eluent.

##### Analysis of reaction products

NMR spectra were recorded on a Brüker Avance 300 spectrometer at room temperature, ^1^H frequency is at 300 MHz, ^13^C frequency is at 75 MHz. IR spectra were recorded on a PerkinElmer Spectrum 100 FT-IR spectrometer, in ATR mode. GC-MS analyses were performed on a Shimadzu QP2010SE apparatus, equipped with a 30 m × 0.25 mm × 0.25 μm ZB-5MSi Guardian column (Phenomenex®) with hydrogen as carrier gas.

##### DFT analysis

Density functional theory (DFT) calculations were performed using the Gaussian 09 computational package (Frisch et al., 2009). The B3LYP functionals was used as implemented in Gaussian. Geometry and electronic structure optimizations were carried out for the functional/basis set combinations using the default optimization algorithm in Gaussian 09. Energy and frequency calculations were performed on fully optimized geometries using the 6-311++G(2d,p) basis set. B3LYP functionals showed their relevance for studying metals (Lai et al., 2012) and it is widely used. Two different models have been applied to determine partial atomic charge: Mulliken (Mulliken, 1955), and Merz–Kollman (MK) (Besler et al., 1990).

## Results and discussion

### Phytoremediation

**Figure 1:**
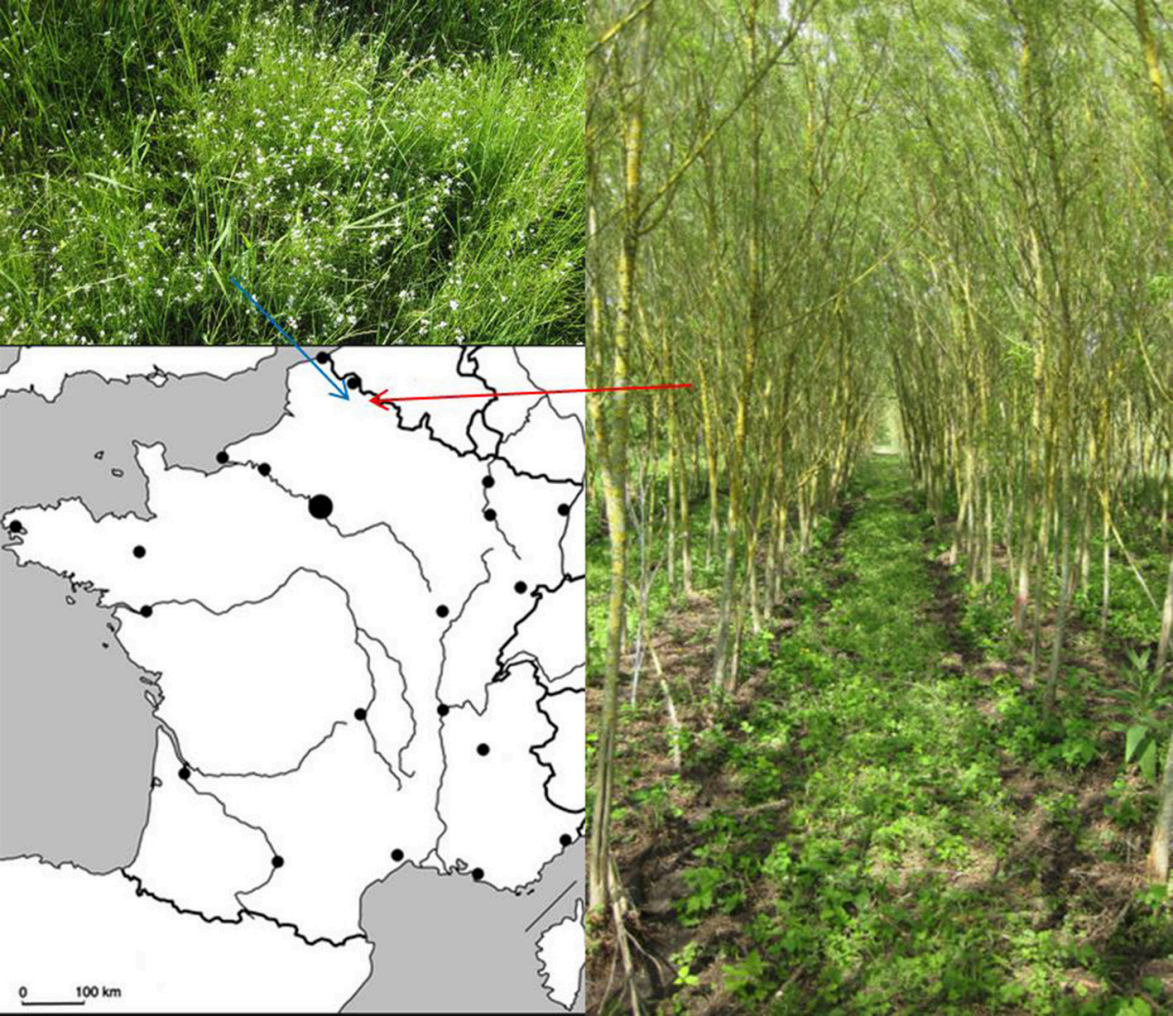
Location of field site projects. *Arabidopsis halleri* (Up-left) in Auby and *Salix “Tordis”* (Right) in Fresnes-sur-Escaut.

In this study, we used a Zn hyperaccumulator plant, *A. halleri*, and a Zn accumulator plant, *Salix “*Tordis.” Both are included in a large program of phytomanagement of metal-contaminated brownfields located in the north of France (Figure 1). After harvesting and drying the leaves of each plant, ICP-AES analyses were realized in order to measure element proportions in the biomass (Table 1). In addition to the normal of physiological species, both plants are able to accumulate Zn, and to a lesser extent Cd. As expected, *A. halleri* hyperaccumulates Zn, that represents more than 1% of its dry weight. The willow confirmed to have a Zn-accumulating behavior as its leaves contained Zn in concentration far above the baseline one. Based on Zn foliar concentrations *A. halleri* and willow leaves are suitable for the Zn-ecocatalyst production.

**Table 1:** ICP-MS Analysis of leaves derived from *Arabidopsis halleri* and *Salix* “Tordis” (ppm).

**Plant**	**As**	**Ca**	**Cd**	**Cr**	**K**	**Mg**	**Mn**	**Ni**	**P**	**Pb**	**S**	**Zn**
*Arabidopsis halleri*	3.05 (1.28)	18,060 (1,266)	141 (40)	1.92 (1.05)	21,180 (3,235)	3,624 (380)	61 (20)	7.64 (2.66)	3,150 (443)	118 (98)	7,570 (698)	12,960 (2,449)
*Salix “Tordis”*	0.28 (0.05)	25,840 (1,857)	9.57 (0.84)	4.33 (1.06)	9,140 (659)	1,780 (94)	63 (5)	1.83 (0.14)	1,739 (99)	3.03 (0.74)	5,690 (407)	3,159 (271)

*Values are means ± SE (in brackets)*.

#### Preparation and characterization of the ecocatalysts

The ecocatalysts were prepared from an important quantity of biomass to homogenize their composition and minimize their variation. The biomass was calcined under air flow to remove most of organic matter (Garel et al., 2015). The mass loss for *A. halleri* and *Salix* was around 80–90%. The residues were treated with hydrochloric acid in order to form active metal chloride salts. According to MP-AES analysis, both ecocatalysts contained zinc in good proportions, accompanied by the physiological metals such as calcium, potassium, and magnesium (Table 2). As expected, the quantity of zinc was higher in the ecocatalyst made from *A. halleri* (Eco-A.h.), a Zn hyperaccumulator (7.6%), than in those derived from *Salix “Tordis”* (Eco-S.T.), a Zn accumulator (2.6%). In comparison with our previous works related to Eco-Zn catalysts, which derived from phytoextraction on mining sites (Grison et al., 2015), the zinc concentration in Eco-A.h. is approximately the same than the one found in the catalyst stemmed from *Noccaea caerulescens*. However, *Anthyllis vulneraria* gave a catalyst with the highest Zn content, better than the two brassicaceae, *A. halleri* and *N. caerulescens* (Table 2). It should be noted that large differences were observed for calcium: the Ca concentration is significantly higher in Eco-S.T. than in Eco-A.h. (respectively, 17.1 and 9.7%).

**Table 2:** MP-AES Analysis of ecocatalysts derived from *Arabidopsis halleri* (Eco-A.h.) and *Salix* “Tordis” (Eco-S.T.) (wt %).

**Ecocatalyst**	**K**	**Mg**	**Ca**	**Zn**
Eco-A. h.	11.8 (± 0.65%)	1.9 (± 4.5%)	12.5 (± 0.51%)	7.6 (± 0.18%)
Eco-S. T.	10.0 (± 0.42%)	1.5 (± 0.78%)	19.8 (± 0.44%)	2.6 (± 1.35%)
Eco-Zn (*Noccaea caerulescens)*	–	1.5 (±0.17%)	–	7.3 (±0.4%)
Eco-Zn (*Anthyllis vulneraria)*	–	3.6 (±0.18%)	–	15.4 (±0.7%)

The two biosourced catalysts were analyzed by X-ray diffraction (XRD). The XRD characterization revealed the presence of an unusual mixture of metal chlorides such as K_2_ZnCl_4_ (potassium tetrachlorozincate), KMgCl_3_·6H_2_O (carnallite), KCl (sylvite), and a remaining sulfate, CaSO_4_·(H_2_O)_0.5_, in the crystalline fraction in both ecocatalysts (Table 3). K_2_ZnCl_4_, a masked form of ZnCl_2_, was observed in Eco-A.h. but not in Eco-S.T. For the latter, zinc was not present in the crystalline phase or at a non-detectable level. Compared to ecocatalysts derived from *N. caerulescens* and *A. vulneraria*, the lack of CaMg_2_Cl_6_·12H_2_O was as surprising as the presence of carnallite (Escande et al., 2014a). Although XRD analyses only testify the presence of crystalline species, eco-catalysts showed a very original composition (see Supplementary Material).

**Table 3:** Crystalline species in ecocatalysts (✔, presence; ✖, lack).

**Catalyst**	**Carnallite KMgCl_3_·6H_2_O**	**Calcium sulfate CaSO_4_·(H_2_O)_0.5_**	**Sylvite KCl**	**Potassium tetrachlorozincate K_2_ZnCl_4_**	**Tachyhydrite CaMg_2_Cl_6_·12H_2_O**
Eco-A.h.	✔	✔	✔	✔	✖
Eco-S.T.	✔	✔	✔	✖	✖

**Figure 2:**
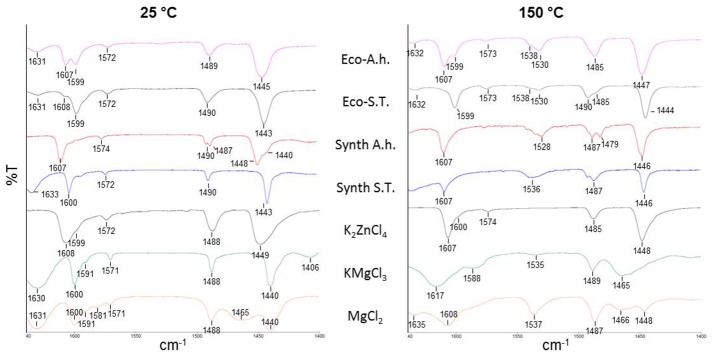
IR spectra of pyridine adsorbed on ecocatalysts (Eco-A.h. and Eco-S.T.), crystalline synthetic salts constitutive of ecocatalysts (Synth-A.h. and Synth-S.T.) and pure salts, recorded after outgassing at 25 and 150°C.

The Lewis acidity of our ecocatalysts has been studied with an empirical method that implies the coordination of adsorbed pyridine on the surface of acidic solids. By studying the bands in the range of 1,640–1,400 cm^−1^, Lewis and Brønsted acidities can be evaluated for each catalyst and compared with classical metal chlorides (Parry, 1963; Zaki et al., 2001). Infrared spectra of pyridine adsorbed on crude fraction of ecocatalysts were recorded at 25°C and after heating at 150°C in order to distinguish the frequencies of physisorbed pyridine from those of pyridine coordinated to Lewis sites (Figure 2). After outgassing at 25°C, IR studies did not show a huge difference between both of the ecocatalysts. Bands at 1,445 cm^−1^ and at 1,443 cm^−1^ for Eco-A.h. and Eco-S.T, respectively, denoted the presence of weakly bonded pyridine and thus Lewis acid sites as well as bands at 1,607 and 1,608 cm^−1^. After outgassing at 150°C, bands at 1,530 and 1,538 cm^−1^ showed the presence of pyridinium ion, which indicates a Lewis acidic character in both ecocatalysts. Eco-A.h. exhibits a band at 1,447 cm^−1^ that is higher than the one in Eco-S.T. (1,444 cm^−1^). The band at 1,607 cm^−1^ is more intense than the one at 1,599 cm^−1^ in Eco-A.h. while it almost disappeared in Eco-S.T. These information led to think that Eco-A.h. possesses a stronger Lewis acidic character than Eco-S.T. This is consistent with the expected acidity of the Zn present in Eco-A.h. compared with Eco-S.T. After degassing at 150°C, very intense bands appear at 1,530–1,538 cm^−1^ for both ecocatalysts. These bands are characteristic of the frequency of the pyridinium ion formed by protonation of the pyridine in the presence of Brønsted acid sites. The two ecocatalyts comprise therefore a Lewis acid character and additionally a Brønsted acid character. This last property was not observed in the case of Eco-Zn which derived from *N. caerulescens* and *A. vulneraria*. In order to understand these original results, a mixture of the synthetic salts identified by XRD for each ecocatalyst was synthetized (Synth-A.h. and Synth-S.T.). Synth-S.T. is composed by KMgCl_3_·6H_2_O, KCl and CaSO_4_·(H_2_O)_0.5_ while Synth-A.h. possessed the same composition with additional K_2_ZnCl_4_. After outgassing at 150°C, major bands of Synth-A.h. at 1,446, 1,528, and 1,607 cm^−1^ are comparable with the original ecocatalyst. The Lewis and Brønsted acidic characters are represented in this mixture of four salts. Concerning Synth-S.T., the Brønsted acidic character is still present whereas the band slightly increased from 1,530 cm^−1^ in Eco-S.T. to 1,536 cm^−1^ in the mixture of salts. However, bands at 1,446 and 1,607 cm^−1^ which are not present in the original catalyst, exhibited a stronger Lewis acidic character. This information led us to think that some salts are amorphous and thus not detectable by XRD analyses. Infrared spectra of adsorbed pyridine on the four pure synthetic salts were also realized. While KCl and CaSO_4_·(H_2_O)_0.5_ did not adsorbed pyridine even after outgassing at 25°C, potassium tetrachlorozincate (K_2_ZnCl_4_), and carnallite (KMgCl_3_·6H_2_O) presented interesting results. Lewis acidity of K_2_ZnCl_4_, was evidenced here by the infrared bands at 1,448 and 1,607 cm^−1^, characteristic of pyridine strongly bonded to Lewis acid sites, observed after outgassing at 25°C. The intensity of this band was persistent after outgassing at 150°C. No Brønsted acidic character was observed. This observation was consistent with this salt's aspect, which is not deliquescent after contact with air compared to ZnCl_2_ and makes it easier to handle. KMgCl_3_·6H_2_O was characterized with the same methodology. Figure 2 shows that the band at 1,400 cm^−1^ observed at 25°C disappeared after outgassing at 150°C. The presence of intense bands at 1,440–1,480 and 1,617 cm^−1^ at 150°C indicated the presence of strongly bonded pyridine. Frequencies of these bands are higher than those observed for K_2_ZnCl_4_ that makes KMgCl_3_·6H_2_O a better Lewis acidity than K_2_ZnCl_4._ The catalytic potential of KMgCl_3_·6H_2_O is little known. To the best of our knowledge, the catalytic activity of KMgCl_3_·6H_2_O is not described in the literature but its potential should be a source of interest. It seemed interesting to compare these results with MgCl_2_, which is most widely used in organic synthesis. At 150°C, MgCl_2_ was characterized by the frequencies of bands at 1,448, 1,608, and 1,635 cm^−1^. The Lewis acidity of MgCl_2_ was therefore lower than the Lewis acidity of KMgCl_3_·6H_2_O. However, unlike K_2_ZnCl_4_, and KMgCl_3_·6H_2_O, the spectrum of MgCl_2_ showed the presence of a very intense band at 1,537 cm^−1^ after degassing at 150°C. This band is characteristic of the presence of Brønsted acid sites, unobserved with KMgCl_3_·6H_2_O. These results highlighted the presence of different acidities in these ecocatalysts.

#### Ecocatalyzed knoevenagel condensations

The catalytic activity of each ecocatalysts was tested using the Knoevenagel condensation, a widely used reaction in organic synthesis. Initial reactions were carried out with benzaldehyde and different nucleophiles using the conditions described for zinc dichloride (Shanthan Rao and Venkataratnam, 1991). Reactions were performed neat, at 100°C, during 1 h and using an amount of ecocatalyst which corresponds to 5 mol% of zinc with respect to aldehyde. Control reactions without catalyst exhibited no conversion of benzaldehyde. Results were compared with commercial ZnCl_2_ and K_2_ZnCl_4_ under same conditions (Table 4).

**Table 4:** GC-MS results of Knoevenagel reactions involving benzaldehyde and several nucleophiles.

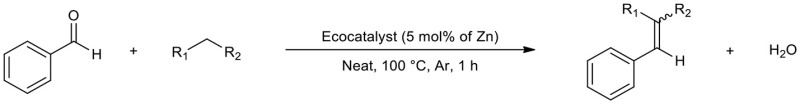										
**Entry**	**R**_1_	**R**_2_	**ZnCl**_2_		**K**_2_**ZnCl**_4_		**Eco-A.h.^b^**		**Eco-S.T.^c^**	
			**Conv.^a^ (%)**	**Select.^a^ (%)**	**Conv.^a^ (%)**	**Select.^a^ (%)**	**Conv.^a^ (%)**	**Select.^a^ (%)**	**Conv.^a^ (%)**	**Select.^a^ (%)**
1	CN	CN	96	>99	85	>99	62 (42)^d^	>99	93 (77)^d^	98
2	CN	CO_2_Me	28 (23)^d^	>99	44	97	53	>99	84	99
3	(CO)CH_3_	(CO)CH_3_	5	–	<5	–	42	72	52	71
4	(CO)CH_3_	CO_2_Et	48	98	0	–	30	73	33	60
5	CO_2_Me	CO_2_Me	0	–	<5	–	10	–	10	–

a Conversion and selectivity determined by GC-MS with biphenyl as internal standard;

b 214 mg of Eco-A.h;

c 614 mg of Eco-S.T.;

d *Knoevenagel product isolated yield*.

Considering these results, the first unexpected trend is that ecocatalysts are more efficient or comparable than pure synthetic salts in most of cases. In the case of methyl cyanoacetate, Eco-A.h. and Eco-S.T. gave 53 and 84% of conversion, respectively, compared to ZnCl_2_ and K_2_ZnCl_4_ which converted benzaldehyde only at 28 and 44% (Table 4, entry 2). Both ecocatalysts have a better ability to convert acetylacetone and dimethylmalonate while pure salts barely reach 5% (Table 4, entries 3 and 5). Interestingly, malononitrile gave a better result by reacting with ZnCl_2_ compared to Eco-A.h. and K_2_ZnCl_4_, but Eco-S.T. provided an excellent conversion in spite of its weak Zn concentration (Table 4, entry 1). The second unexpected trend is that the catalytic behavior of K_2_ZnCl_4_ is disappointing. Except for the special case of malononitrile, K_2_ZnCl_4_ provided lower conversions than Eco-A.h. According to these results, the presence of this salt in Eco-A.h. cannot explain the catalytic efficiency of this ecocatalyst. The third observation is closely linked to the second one: Eco-S.T. which is less concentrated in Zn than Eco-A.h. gave better conversions in four cases (Table 4, entries 1–4) and was comparable in the last case (Table 4, entry 5). Eco-S.T. did not exhibit K_2_ZnCl_4_ in XRD analyses but this ecocatalyst provided better results. There is every reason to believe that the active species is not K_2_ZnCl_4_. In terms of selectivity, reactions only gave Knoevenagel product excepted for entries 3 and 4. These formed products possess a α,β-unsaturated carbonyl group which can undergo a Michael reaction with another equivalent of nucleophile when the reaction time is extended. To check the range of applicability of these ecocatalysts and the electronic effect of different substituents, the reaction was extended to other benzaldehyde derivatives with acetylacetone as nucleophile (Table 5). The results obtained in Table 5 were coherent with the electronic effects of the substituent on the reaction mechanism. As expected, donor groups lower the conversion (Table 5, entries 1 and 2) whereas attractor groups increased it (Table 5, entries 3 and 4). Additionally, Eco-S.T. led to better results than Eco-A.h. in all cases. This suggested that ZnCl_2_ and K_2_ZnCl_4_, and therefore zinc salts, were not the active species during the catalytic process. Infrared studies in Figure 2 revealed a significant Lewis acidity for KMgCl_3_·6H_2_O which was detected in both ecocatalysts by XRD analysis. This salt was thus synthesized in order to be tested. The amount of potassium magnesium trichloride was calculated by considering the magnesium content of Eco-S.T. Results are summarized in Table 6. The three catalytic systems are active in the Knoevenagel condensations of benzaldehyde with various nucleophiles. In most of cases, KMgCl_3_·6H_2_O gave results which are closer to those obtained with Eco-S.T. than MgCl_2_ or zinc salts (Table 6, entries 1–3). According to these encouraging results, it is clear that KMgCl_3_·6H_2_O and its strong Lewis acidic character play a significant role into the catalytic activity applied to the Knoevenagel condensation. It is also important to consider the great interest of using KMgCl_3_·6H_2_O in organic synthesis. Indeed, carnallite is a natural ore which the source of magnesium and potassium for industrial needs. This potentiality of the novel Lewis acid in green catalysis required complementary study.

**Table 5:** GC-MS results of Knoevenagel condensation of *p*-substituted benzaldehyde with acetylacetone as nucleophile.

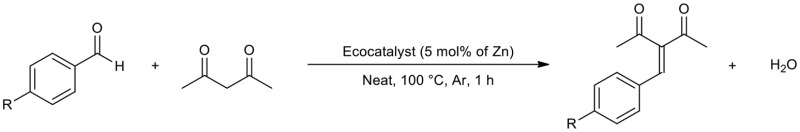					
**Entry**	**R**	**Eco-A.h.^b^**		**Eco-S.T.^c^**	
		**Conv.^a^ (%)**	**Select.^a^ (%)**	**Conv.^a^(%)**	**Select.^a^ (%)**
1	OCH_3_	14	–	30	99
2	CH_3_	26	98	43	99
3	Cl	42	99	64	99
4	NO_2_	36	94	74	99

a Conversion determined by GC-MS with biphenyl as internal standard;

b 214 mg of Eco-A.h.;

c *614 mg of Eco-S.T*.

**Table 6:** GC-MS results of Knoevenagel condensations of benzaldehyde with different nucleophiles catalyzed by magnesium salts.

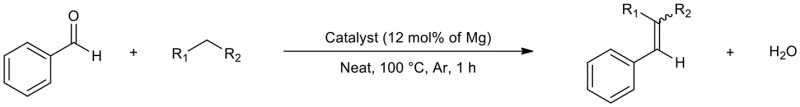								
**Entry**	**R**_1_	**R**_2_	**KMgCl_3_^b^**		**MgCl_2_^c^**		**Eco-S.T.^d^**	
			**Conv.^a^ (%)**	**Select.^a^ (%)**	**Conv.^a^ (%)**	**Select.^a^ (%)**	**Conv.^a^ (%)**	**Select.^a^ (%)**
1	CN	CN	70	>99	61	>99	93 (77)^e^	98
2	CN	CO_2_Me	92	>99	81	>99	84	99
3	(CO)CH_3_	CO_2_Et	59	65	55	82	33	60
4	CO_2_Me	CO_2_Me	22	96	41	98	10	–

a Conversion determined by GC-MS with biphenyl as internal standard;

b 112 mg of KMgCl_3_;

c 60 mg of MgCl_2_;

d 614 mg of Eco-S.T.;

e *Knoevenagel product isolated yield*.

### DFT analysis of catalytic salts and discussion

In view of the results obtained with KMgCl_3_ in the Knoevenagel condensations, it seemed interesting to compare the reactivity of MgCl_2_ and KMgCl_3_. For this, we relied on the concept of Gutmann (Staemmler, 1979; Jensen, 1980), which has been supplemented by Denmark (Denmark and Wynn, 2001). According to Gutmann, the formation of an acid-base adduct induces an overall increase in the electron density in the acceptor fragment of the adduct. Similarly, Denmark, then Massa (Massa et al., 2010) demonstrated that a low Lewis acid, SiCl_4_, can be activated by a Lewis base to promote allylation and propargylation of aldehydes. Similarly, Marcantoni described a surprising catalytic activity for the CeCl_3_-NaI system (Bartoli et al., 2006). According to the concept pushed forward by Gutmann, Denmark and the observations of Marcantoni, we can anticipate an interesting reactivity of KMgCl_3_ driven by an increasing positive charge of Mg. Indeed, the binding of an additional chloride could enhance the electrophilicity character of the Mg. In this hypothesis, KMgCl_3_ should be a better Lewis acid than MgCl_2_, which is supported by IR data. We performed DFT calculations to evaluate the electronic redistribution resulting of an additional chloride in KMgCl_3_ in comparison with MgCl_2_. As shown by the other experiments, the potassium (K^+^) does not seem to interfere in the reactivity. The calculations have therefore been performed without the K^+^ counterion. Furthermore, as it is presumed that the metal is interacting with the benzaldehyde carbonyl during the catalytic process, another set of experiments assesses the electron density of the metal in presence of benzaldehyde (BA). All the experiments were performed in the gas phase (see Experimental section for details). The results are shown in Table 7. The Mulliken population analysis is often considered as fair standard to evaluate the atomic charge, but it suffers a lot from basis set dependency. On the contrary the Merz-Singh-Kollman (MK) charges are known as being rather basis independent. In both cases, there is a clear tendency that can be drawn: the Mg atom is more electropositive when holding an extra Cl. This nicely converges with the Gutmann analysis and the experimental data showing that the ${\text{MgCl}}_{3}^{-}$ is more reactive than MgCl_2_.

**Table 7:** Summary of Mulliken, MK charges, and softness on Mg assessed by DFT calculations performed using Gaussian09.

	**MgCl_2_**	**${\text{MgCl}}_{3}^{-}$**	**BA–MgCl_2_**	**BA–${\text{MgCl}}_{3}^{-}$**
Mulliken charge on Mg^a^	0.6505	0.996948	0.8083	1.1180
MK charge on Mg^a^	0.8357	0.8382	0.7978	0.8809
Softness^b^	4.155	4.351	6.942	8.946

a Calculated at the B3LYP/6311-G(2d,p) level of theory;

b *Used for the HSAB concept (Hard Soft Acids and Bases), also known as the Pearson acid base concept (Pearson, 1968), the hardness () and softness (S) can be estimated using the following formulas (Pearson, 2005): η ≈ E_LUMO_ − E_HOMO_ and $S\approx \frac{1}{\eta }$ where E_LUMO_ = lowest unoccupied molecular orbital energy and E_HOMO_ = highest occupied molecular orbital energy*.

## General conclusion

The aim of this work was basically to determine if the utilization of the Zn-accumulating plant *Salix Tordis* and the hyperaccumulating plant *A. halleri* was valuable in catalysis. To answer this question, two ecocatalysts were synthesized from biomass used in phytomanagement, in order to be tested in the Knoevenagel condensation reaction. Both ecocatalysts were able to convert aldehydes but interestingly, the conversion promoted by Eco-S.T. was better than with Eco-A.h. in spite of its lower zinc concentration. While our hypotheses were focused on zinc salt to act as the active specie during the catalytic process, the surprising activity of the magnesium salt KMgCl_3_ was out of our expectations. The results obtained in catalysis with this salt confirmed its strong Lewis acid character which was first observed in infrared studies and also its remarkable role to convert benzaldehyde compared to K_2_ZnCl_4_ and MgCl_2_. These observations were supported by DFT calculations which showed that magnesium is a better Lewis acid when it possesses three chlorine atoms in its first coordination sphere instead of two. With these experimental results, we were able to show the relevance of both Zn-accumulating plants. Finally, these new bio-based polymetallic catalysts illustrated the interest of this concept for green and sustainable catalysis.

## Author contributions

CG is the principal investigator of the work. P-AD carried out the organic synthesis. VB is responsible for the phytomanagement part. Y-ML and EP are responsible for the DFT calculation. CG and P-AD are responsible for the writing. CB is responsible for XRD analyses. SD realized the XRD analyses.

### Conflict of interest statement

The authors declare that the research was conducted in the absence of any commercial or financial relationships that could be construed as a potential conflict of interest.
