# Supplementary material for: Biosourced Polymetallic Catalysis: A Surprising and Efficient Means to Promote the Knoevenagel Condensation
Source: Front Chem. 2018 Mar 27;6:48. doi: 10.3389/fchem.2018.00048 (PMC5881248; doi:10.3389/fchem.2018.00048)
Supplement: Supplementary file 1 [file DataSheet1.docx]

Supplementary Material

Biosourced polymetallic catalysis: A surprising and efficient means to promote the Knoevenagel condensation

Pierre-Alexandre Deyris^a^, Eddy Petit^b^, Yves-Marie Legrand^b^, Sébastien Diliberto^c^, Clotilde Boulanger^c^, Valérie Bert^d^, Claude Grison^a*^

*** Correspondence:** Claude Grison. [claude.grison@cnrs.f](mailto:claude.grison@cnrs.f)

# Supplementary Figures


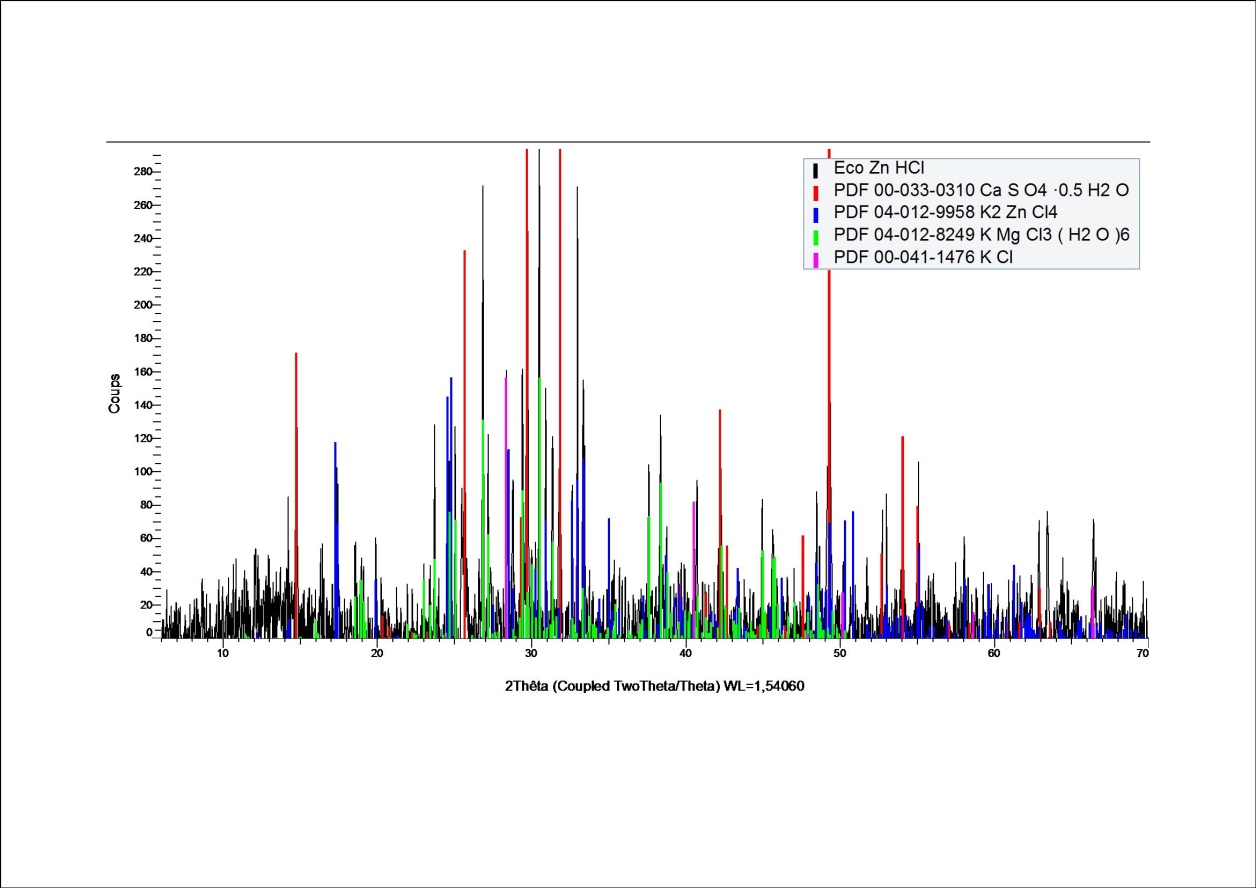


**Supplementary figure 1**: XRD analyses of ecocatalyst derived from *Arabidopsis halleri* (Eco-A.h.) Black: Eco-A.h.. Red : calcium sulfate. Blue : potassium tetrachlorozincate. Green : carnallite (KMgCl_3_·6H_2_O). Violet : Potassium chloride.


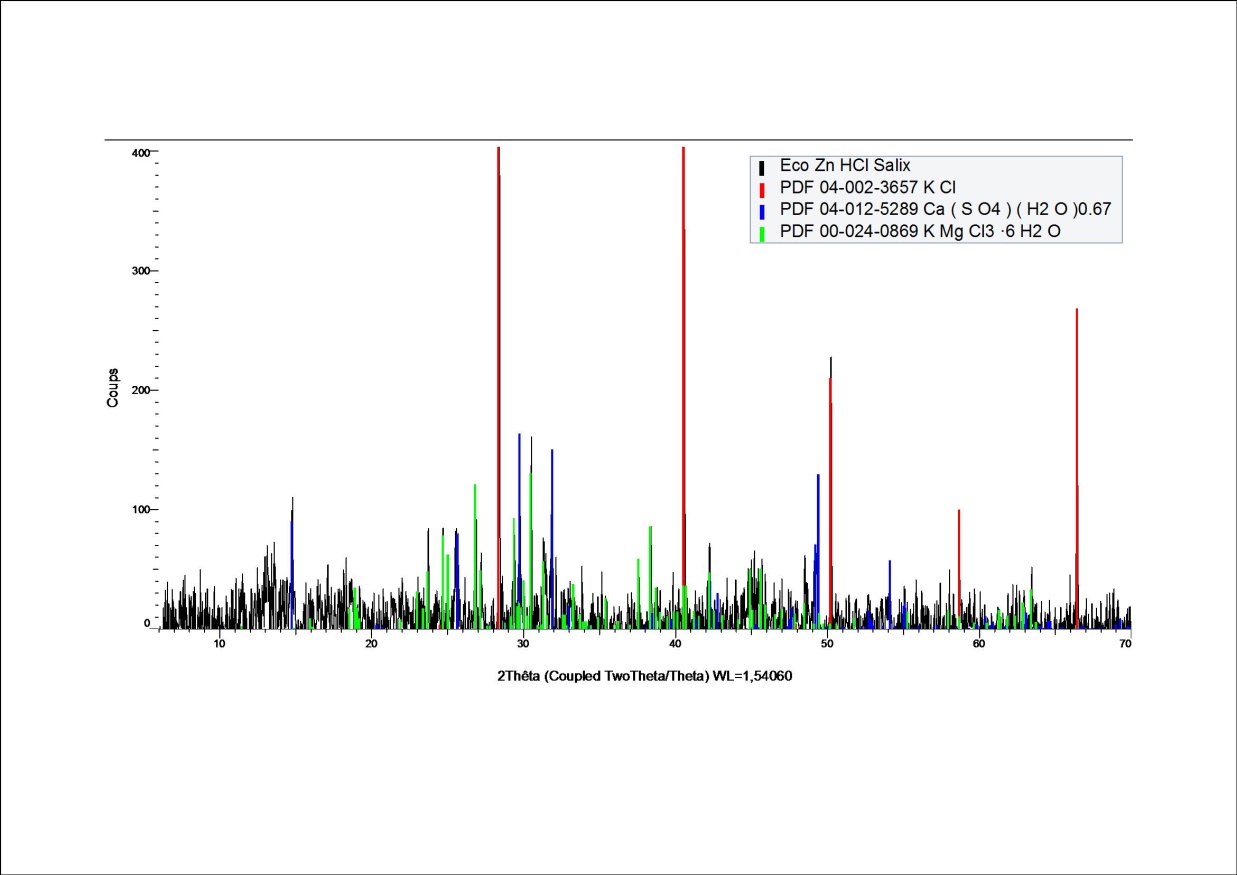


Supplementary figure 2: XRD analyses of ecocatalyst derived from Salix ‘Tordis’ (Eco-S.T.) Black: Eco-S.T.. Red : Potassium chloride. Blue : calcium sulfate. Green : carnallite (KMgCl3·6H2O).
